# Fabrication of Ti_3_Al-Based Intermetallic Alloy by Laser Powder Bed Fusion Using a Powder Mixture

**DOI:** 10.3390/ma16072699

**Published:** 2023-03-28

**Authors:** Kuanhe Li, Xianglong Wang, Haishao Chen, Xiaoxiao Huang, Guanglin Zhu, Ganfeng Tu

**Affiliations:** 1 School of Metallurgy, Northeastern University, Shenyang 110819, China; 2 China Machinery Institute of Advanced Materials Co., Ltd., Zhengzhou 450001, China; 3 State Key Laboratory for Advanced Forming Technology and Equipment, China Academy of Machinery Science and Technology, Beijing 100083, China; 4 Mining and Materials Engineering, McGill University, 3610 University Street, Montreal, QC H3A 0C5, Canada; 5 School of Intelligent Manufacturing and Electronic Engineering, Wenzhou University of Technology, Wenzhou 325025, China

**Keywords:** laser powder bed fusion, additive manufacturing, Ti_3_Al alloy, intermetallic alloy

## Abstract

Due to their light weight and outstanding mechanical properties at high temperatures, Ti_3_Al-based intermetallic alloys have driven increasing interest from both academia and industry; however, when additive manufacturing (AM) is applied to them, the outcome is hardly satisfying. In this work, we report a crack-free Ti_3_Al-based alloy fabrication by laser powder bed fusion (LPBF) using a mixture of a commercial Ti-48Al-2Cr-2Nb powder and a pure Ti powder. With the aid of a high cooling rate during LPBF, the as-built sample shows a ductile β phase with some partially-melted particles. After the heat treatment, partially-melted particles were dissolved, and the sample showed equiaxed α_2_ precipitates in the β matrix. The hardness was 515 ± 38 HV in the as-built sample and 475 ± 37 HV in the heat-treated sample. This study shows a novel strategy to fabricate crack-free Ti_3_Al-based alloy using LPBF from powder blends.

## 1. Introduction

Over the last 30 years, titanium aluminide intermetallic alloys, based on TiAl and Ti_3_Al, have driven plenty of interest in high-temperature applications up to 650 °C, including turbine blades, engine components, etc. [1]. Compared to Ni superalloys and Ti alloys, titanium aluminide alloys possess various advantages at high temperatures, such as low density, outstanding strength, superior creep resistance, and good corrosion resistance, and are thus considered to be a promising candidate material for the next-generation superalloy [2,3].

As an advanced manufacturing technology, additive manufacturing (AM) has won growing attention, due to its advantages in complex lattice structure fabrication, near-net-shape production, and short lead time customization [4,5,6]. Hence, applying AM to titanium aluminide alloys has emerged as a research hotspot [7,8,9,10,11]. However, the sourcing of the powders is hindering the development of some material systems, including Ti_3_Al alloys, due to the lack of commercialized powder and the high cost of customizing the pre-alloyed powders [12,13]. Therefore, LPBF with a powder blend would be an alternative method. Nevertheless, compositional homogeneity is the major concern of using powder blends for LPBF. The melting point difference of the powder ingredients apparently increases the possibility of inhomogeneity. Recently, some attempts were carried out based on powder blends, such as NiTi [14], Ti6Al4V [15,16], Fe-Cr-Ni [17], etc. However, some high-melt elements, e.g., Nb in the Ti2AlNb system [18], can significantly deteriorate the homogeneity of the material [19]. In this study, an assumption based on powder blends of commercially pure Ti (CP-Ti) and TiAl-4822 has been proposed. As nearly the only commercialized titanium aluminide alloy, TiAl-4822 and its powders, with a nominal composition of Ti-48Al-2Cr-2Nb (at.%), can be easily sourced. The pre-alloyed powders naturally prevent the inhomogeneity of high-melt Nb. Additionally, the TiAl-4822 powders have a melting point of 1490 °C, similar to the CP-Ti powder (1668 °C). Therefore, the compositional homogeneity can be good and the difficulty of the following heat treatment (HT) can be reduced. 

On the other hand, cracking is also a major barrier to achieving the fabrication of satisfying titanium aluminum alloys by AM [20,21,22]. The intrinsic brittleness of titanium aluminum alloys, accompanied by the rapid solidification and high residual stresses in most metal AM, leads to the cracking problem [23,24,25]. Ti_3_Al-based alloys suffer from low ductility at ambient temperature, primarily due to the high-volume fraction of the hcp α_2_ phase (D0_19_). This ordered hexagonal structure provides limited slip systems during deformation, which are not enough to sufficiently accommodates dislocation slip, thus resulting in the inherent brittleness of Ti_3_Al-based alloys at room temperature [26]. To deal with this drawback, the introduction of the ductile β phase is considered to be a possible solution because it features a symmetric cubic structure with sufficient slip systems. The presence of the β phase at room temperature can be achieved in Ti_3_Al alloys, mainly by adding β-solidifying elements such as Mo, Nb, V, and Cr [1,27]. Therefore, based on the β/α_2_ duplex structure, several alloy compositions such as super α_2_ alloy (Ti-25Al-10Nb–3V–1Mo, at.%) [28,29], Ti-24Al-11Nb (at.%) [30], etc., have been investigated and developed. In these studies, however, high volume fractions of β phase have not been obtained, whereas the high amount of Nb addition also significantly increases the density and production cost of the alloys. Recently, some research has reported that unique phase constitutions may be formed due to the rapid solidification conditions of metal AM processes, which may introduce out-of-equilibrium phases (e.g., β phase), providing an alternative way of controlling phase constituents other than the addition of alloying elements. For example, the disordered β phase was achieved using laser powder bed fusion (LPBF) to increase the ductility in a Fe-Co intermetallic system [31]. Durejko et al. [32] also showed the modulation of D0_3_/B2 phase proportion in an Fe_3_Al system by controlling AM parameters. Our previous study suggested that, with the aid of a high cooling rate in LPBF, TiAl-4822 showed a high fraction of β phase [33]. The Ti_3_Al alloy undergoes a wider β phase field during cooling, and thus a high fraction of disordered β can be expected, which secures the components from the initial cold cracking. 

In this study, the Ti_3_Al-based alloy was processed by LPBF from a CP-Ti and TiAl-4822 powder blend. The crack-free thin strut sample has been accomplished as fundamental research for the AM-aided complexed lattice structure. The phases, microstructure, and texture evolution were characterized by X-ray diffraction (XRD), scanning electron microscope (SEM), electron dispersive spectrometer (EDS), and electron backscattered diffraction (EBSD). After the HT, the partially-melted particles were fully dissolved, and an α_2_/β duplex structure was achieved. The microhardness was also investigated to show the mechanical property. This study shows a novel strategy to process Ti_3_Al-based alloy using LPBF from powder blends.

## 2. Materials and Methods

The powder feedstocks were Ti-48Al-2Cr-2Nb (TiAl-4822) powder and pure Ti powder. The commercial TiAl-4822 powder was sourced from AP&C Company, with a nominal composition of Ti-48% Al-2% Nb-2%Cr (at.%). Pure Cp-Ti powder was also sourced from AP&C Company. The powder blend consisted of 56 g CP-Ti powders for every 100 g TiAl-4822 powder. The powders were mixed and homogenized in a tumbler mixer for 4 h. A rolling mill drives the tumbler container to rotate, with the powder blend in, and in this way the powder blend was mixed. The powder size distribution was characterized by an LA-920 Horiba laser particle size analyzer, and the morphology and chemical composition were analyzed using a Hitachi SU3500 SEM with an EDS. For phase characterization, a Bruker D8 Discovery X-ray diffractometer with a Cu radiation source was used under a 2θ range of 15–100 degrees. 

Strut samples with a nominal diameter of 1 mm were fabricated, using a customized LPBF system equipped with a pulsed Ytterbium fiber laser having a maximum power output of 25 W in a pure Ar atmosphere. The experimental parameters were as follows: The laser beam focus diameter is 110 µm, the laser scanning speed is 150 mm/min, the laser frequency is 25 pulses per second, the exposure time of each pulse is 0.8 ms, the hatch spacing is 100 µm, and the layer thickness is 50 µm. The substrates used in the experiments were commercial Ti-6Al-4V plates. HT at 1185 °C for 4 h was applied to the as-built samples.

Before metallurgical characterization, the as-built and HT samples were sectioned along the transverse direction, followed by sequential grinding steps up to 800 grit using SiC grinding papers. The polishing steps were done using 9 μm, 3 μm, and 1 μm diamond suspensions. Finally, the samples were polished with 0.05 µm colloidal silica suspension in a Vibromet 2 machine for 30 h. 

To identify the phase of the as-built and HT samples, the Cu source Bruker D8 XRD was used, where the scans were carried out within a 2θ range of 15 to 100 degrees with a step size of 0.005 degrees. To reveal the microstructure, a Keyence VHX-S550E optical microscope and the Hitachi SU3500 SEM equipped with an EDS detector were utilized. For the crystallographic texture analysis, an EBSD system mounted on the Hitachi SU3500 SEM was utilized. The acquisition was conducted at an accelerating voltage of 15 keV and a step size of 2.2 μm and 0.5 μm. After the acquisition, the raw data were processed with the HKL Channel 5 software.

To investigate the mechanical properties, Vickers microhardness measurement was conducted with a CM-100AT Clark Microhardness Indenter to obtain hardness profiles of the samples. The tests were performed at a load of 300 gf.

## 3. Results and Discussion

### 3.1. Powder Mixture Characterization

**Figure 1:**
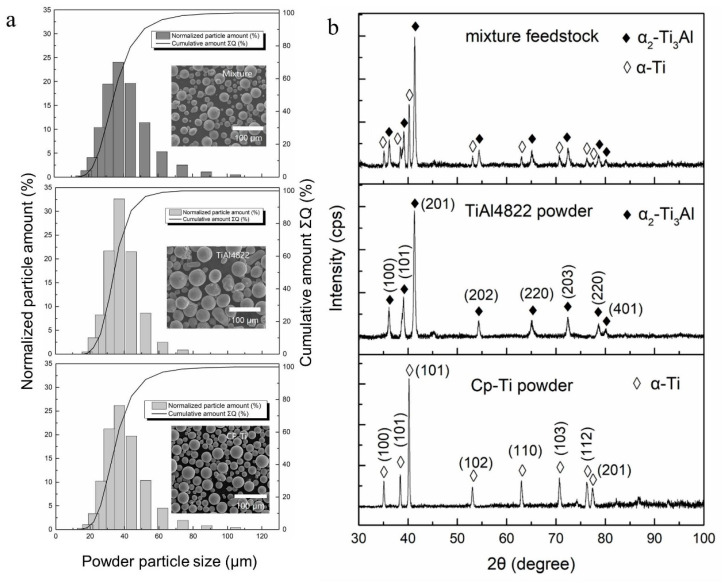
(**a**) Histogram of powder size distribution and the corresponding powder morphologies of the powder mixture, TiAl-4822, and Cp-Ti; (**b**) XRD patterns of the mixture, TiAl-4822, and Cp-Ti powders.

The powder morphologies of the powder mixture, TiAl-4822, and CP-Ti were shown in Figure 1a, indicating similar spherical shapes and smooth surfaces. The selected TiAl-4822 and the CP-Ti also showed close powder size distributions, as shown in Figure 1b. The D_10_, D_50_, and D_90_ of TiAl-4822, CP-Ti, and the mixture are shown in Table 1.

**Figure 2:**
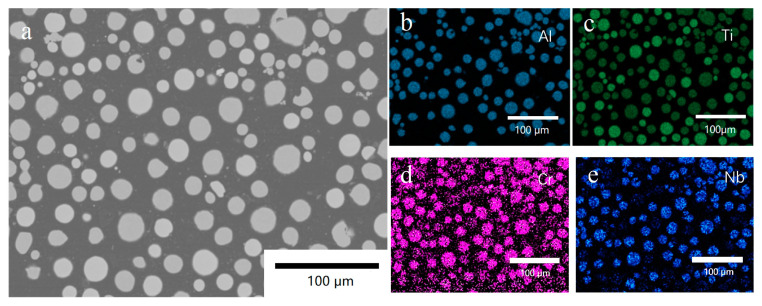
(**a**) BSE image of the cross-section of powder mixture from TiAl4822 and pure Ti powders, and EDS map of (**b**) Al, (**c**) Ti, (**d**) Cr, (**e**) Nb.

Figure 2a–e show the BSE image on the powder’s cross-section and the corresponding EDS maps of Al, Ti, Cr, and Nb, respectively. Figure 2a highlights a large fraction of spherical particles with low porosity. It is worth noting that Figure 2c contains some darker green regions, indicating a relatively lower Ti content, which corresponds well with the contrasts in the EDS maps of Al, Cr, and Nb in Figure 2b,d,e. Therefore, these regions represent the TiAl-4822 powders. On the other hand, the brighter green regions in Figure 2c were identified as CP-Ti powder since the same regions did not show Al, Cr, and Nb signals. The EDS maps indicated that a homogeneous mixture was achieved and was qualified to be the feedstock for LPBF.

### 3.2. Characterization of the As-Built Sample

**Figure 3:**
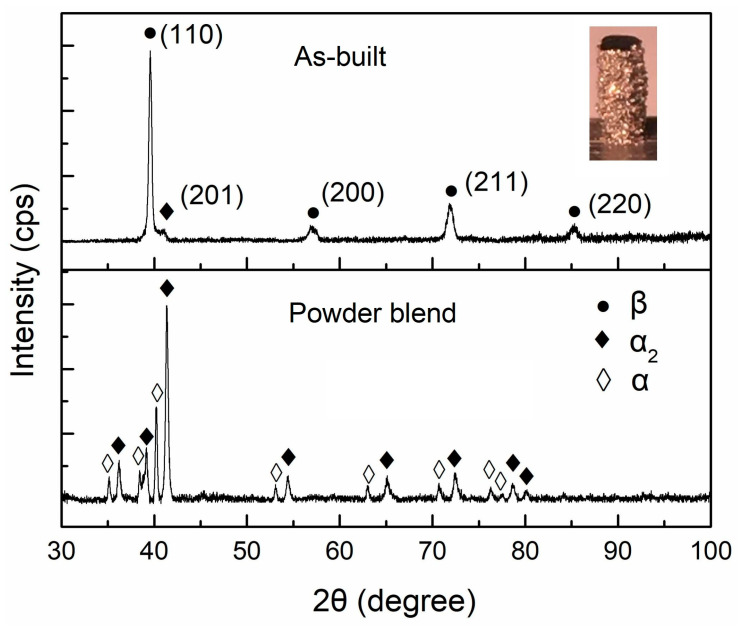
XRD patterns of the as-built sample and powder mixture feedstock, with an inserted picture showing the appearance of the rod sample prepared by LPBF.

The XRD patterns of the as-built sample and powder mixture feedstock are shown in Figure 3, with the thin strut sample fabricated by LPBF inserted. Compared with the XRD pattern of the powder mixture of CP-Ti and TiAl-4822, the as-built sample consisted of nearly a single β phase. No large fraction of the α phase from CP-Ti was detected. This may contribute to the small amount of unmelted CP-Ti powder, whose major peak is at 40.161° (JCPDS: 65-9622) and coincides with the (100) β peak. The presence of these particles will be discussed using BSE and EBSD characterization later. A small amount of α_2_ phase can be confirmed by the diffraction peak at 41.035° (JCPDS: 65-7534). This small amount of α_2_ phase can be considered mainly as the partially-melted TiAl-4822 in the heat-affect-zone. In the β-solidifying Ti_3_Al alloys, the transformation from disordered β to its ordered counterpart β_0_ (B2) has been widely reported [34,35]. This ordered phase is regarded as detrimental to room-temperature ductility [35]. The switch of lattice parameters from 0.3206 nm to 0.3186 nm is a criterion to identify the disordered β phase and ordered β_0_ phase. The small number of alloying elements provides limited influence on the lattice parameters, typically at the order of 0.0001 nm. Based on the XRD pattern, the lattice parameter was calculated to be 0.3208 nm, matching with the β phase lattice parameter reported by Holec et al. [34], implying the presence of a high fraction of disordered β phase in the as-built sample. In addition, with the increase of Nb, the ordering temperature of β/B2, increases [36], which suppresses the ordering transition and increases the possibility of β phase. Essentially, the high cooling rate (at the level of 10^5^–10^7^ K/s [23,37]) generated by the pulsed laser facilitates the formation of the β phase. The disordered β is intrinsically ductile and could accommodate the potential cracking propagation, securing the components from the initial cracking [33]. It should be also noted that the thin strut structure has relatively low residual stress in the LPBF fabrication. To scale up the strut component, more investigation is also required to control the content and distribution of the β phase and the partially-melted particles to resist the negative effect of scaling up. 

**Figure 4:**
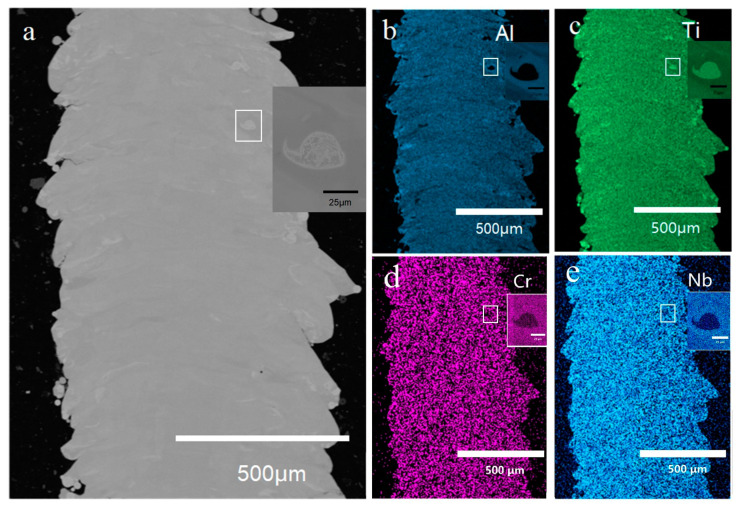
(**a**) BSE image of the as-built sample cross-section by LPBF, (**b**) EDS map of Al, (**c**) map of Ti, (**d**) map of Cr, (**e**) map of Nb.

BSE image of the as-built sample was shown in Figure 4a without showing any cracks. This phenomenon gives an optimistic expectation in the Ti_3_Al complex lattice structure manufacturing in the future. One of the concerns of the powder mixture LPBF is the sufficiency of the melting and the chemical homogeneity. The EDS maps in Figure 4b–e show that the as-built sample had a comparably good homogeneity, despite some partially-melted particles. Some of the particles have a thin plate-like shape, and others showed a near-spherical shape. The relative density of the partially-melted particles is ~8% in total, and the unmelted particles were more frequently observed near the edge of the strut sample. A partially-melted particle was shown using higher magnification in Figure 4a, and its corresponding EDS map is shown in Figure 4b–e. 

**Figure 5:**
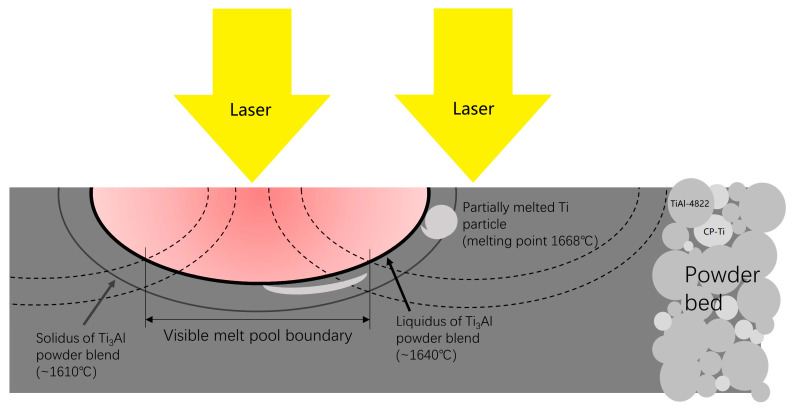
Schematic showing the formation of the partially-melted particles.

A schematic in Figure 5 illustrates the presence of partially-melted Ti particles in the as-built sample. CP-Ti has a higher melting point (1668 °C) than both TiAl-4822 (~1490 °C) [38] and the idealistic Ti_3_Al mixture (~1610 °C). In the center of the melt pool, the temperature is high enough, and thus it is sufficient to obtain a well-stirred mixture. However, the heat-affected zone is below the liquidus of the Ti_3_Al mixture at ~1610 °C and above the solidus at ~1640 °C. When the Ti particle happens to be on the edge of the heat-affected zones, the melting can be insufficient, and the particles were sintered. Some of the partially-melted particles are located near the surface, which can be remelted by the overlapping of the next melt pool (shown in the dashed line). In the peripheral region of the sample, it was not as possible to sufficiently remelt it. Therefore, the number of partially-melted particles increased. If the partially-melted particles were at the bottom of the heat-affected zone, they could not be remelted by the overlapping. In the same scenario, TiAl-4822 particles can also be sintered. As a result, partially-melted particles were observed.

**Figure 6:**
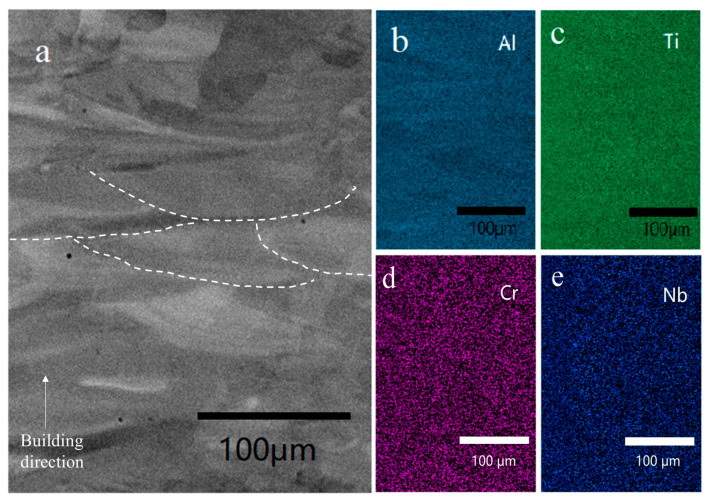
(**a**) BSE image of the central region of the as-built sample, and EDS maps of (**b**) Al, (**c**) Ti, (**d**) Cr, and (**e**) Nb.

The BSE image in Figure 6a shows a typical central region of the as-built sample, with the melt pool represented by the dashed lines. Based on the EDS maps shown in Figure 6 b–e, Al segregation was observed associated with the melt pool shapes. The lack-of-Al regions were found along the melt pool boundaries. This phenomenon can be explained by the continuous growth model (CGM) developed by Aziz et al. [39]. In a non-equilibrium solidification, with the increase of the solidification front velocity, the solute partitioning effect is largely different from the equilibrium. At the bottom of a melt pool, the solidification front velocity is relatively lower, and Al segregates generally following the equilibrium phase diagram. With the increase of the solidification velocity, the segregation becomes insufficient, and Al content piles up to the melts. Therefore, the central region of a melt pool has a relatively higher Al content, and the Al-lack region corresponds to the bottom of the melt pools.

**Figure 7:**
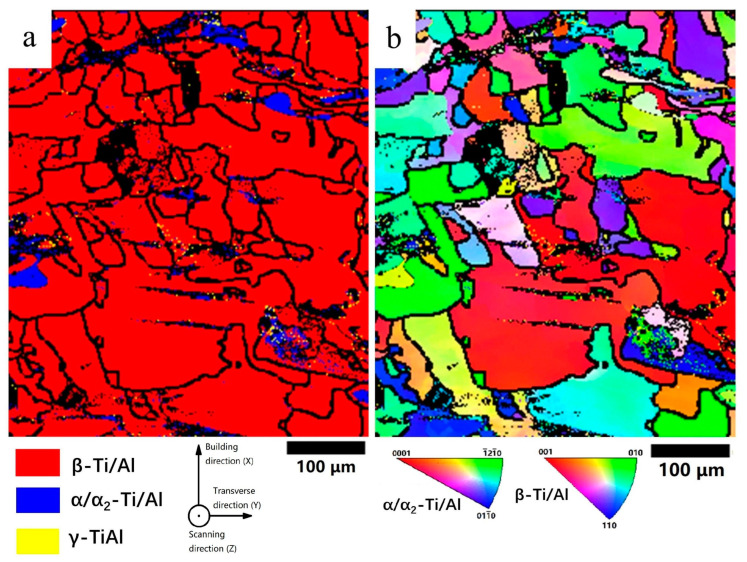
(**a**) EBSD colored phase map and (**b**) inverse pole figure colored map of the as-built sample.

To further understand the solidification process and the microstructure, EBSD was applied, and the results are shown in Figure 7. Figure 7a is the phase map, indicating that the as-built sample has a large fraction of β phase and some partially-melted particles dispersed in the β matrix. The fraction of β phase was measured to be 94.7%. With the aid of the ductile β phase, the intrinsically brittle Ti_3_Al could be fabricated without cracks, regardless of the negative effect of high residual stress. It should be noticed that EBSD can not differentiate α and α_2_ phases in this condition, and thus the partially-melted particles could be either of them. Figure 7b is the inverse pole figure. The shape of the β grains was irregular and asymmetrical, and some large grains grow through multiple layers, showing a sign of epitaxial growth. The epitaxial growth during LPBF typically follows the maximum heat flow direction based on the local curvature of the melt pools [23]. In this study, the partially-melted particles can affect the curvature of a melt pool and the grain growth direction. 

### 3.3. Characterization of the Heat-Treated Sample

**Figure 8:**
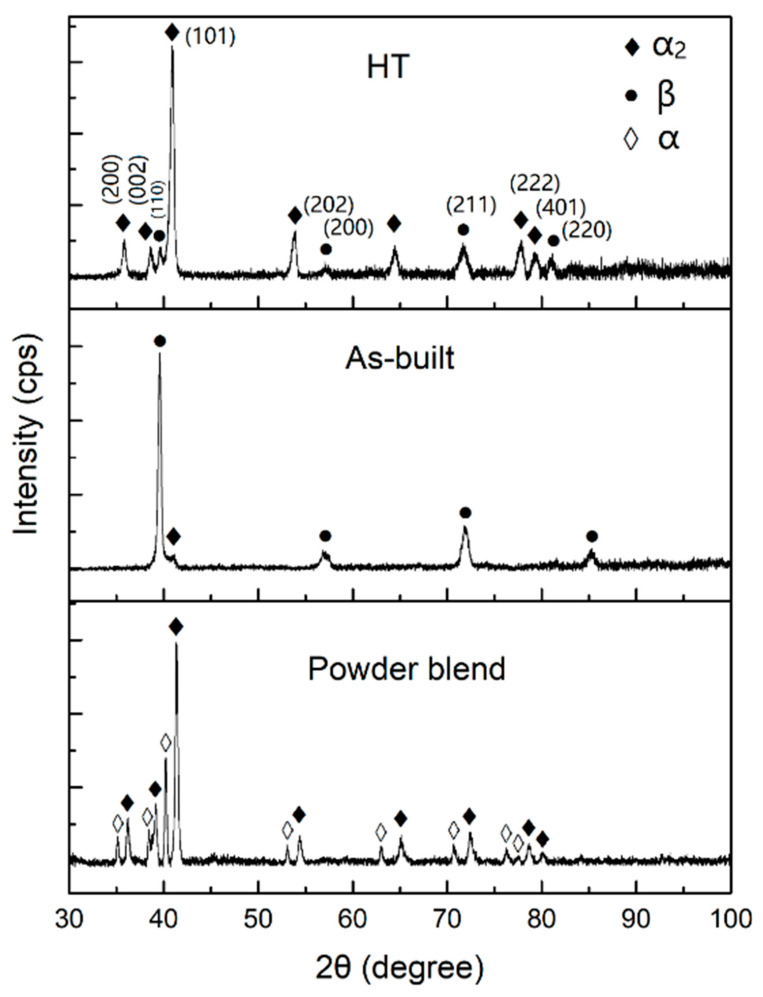
XRD patterns of the as-built sample and powder mixture feedstock.

To achieve a satisfying homogeneity, an HT at 1200 °C (β single phase region) for 4 h, followed by air cooling, was applied. With the aid of XRD, phase evolution was revealed, as shown in Figure 8. Compared with the as-built sample, the HT sample contained a higher fraction of α_2_ phase, which was very low in the as-built sample. The β phase fraction decreases in the HT sample, and the lattice parameter of the β phase was 0.32062 nm, which indicates that it remains a disordered β phase. This phenomenon is different from some previous studies, where the ordering transition from β to B2 takes place during air cooling [40,41,42]. This phenomenon can be attributed to the low Nb fraction at ~2 wt.%. In the pseudo-binary phase diagram of Ti_3_Al-Nb by Strychor [43], the low Nb region maintains the β phase instead of the B2 phase. The study on Ti-25.2 at.% Al by differential thermal analysis and differential scanning calorimetry also support this understanding [44]. The β phase is considered to be beneficial to ductility and deformability, which is crucial to controlling the cracking susceptibility during AM. It can be expected that the Ti_3_Al alloy with a low Nb content can be a promising candidate in the AM society.

**Figure 9:**
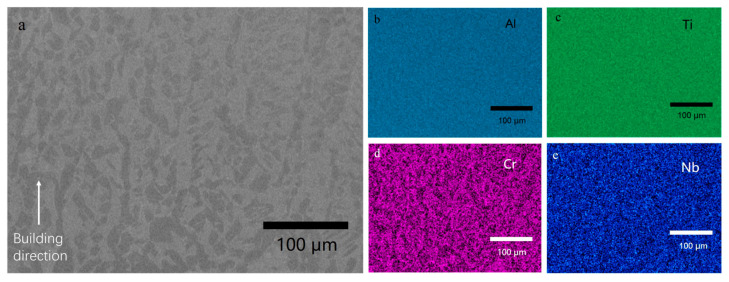
(**a**) BSE image of the HT sample, and EDS map of (**b**) Al, (**c**) Ti, (**d**) Cr, (**e**) Nb.

As shown in Figure 9a, the BSE image of the HT sample shows no partially melted particles. The fine recrystallized α_2_-Ti_3_Al equiaxed grains were observed in a darker contrast dispersed in the β phase matrix. Some of the equiaxed α_2_ grains aligned in a row, along the building direction. The equiaxed α_2_ structure has been reported [45]. The EDS maps of Al, Ti, Cr, and Nb are shown in Figure 9b–e. The Al and Ti distributions show a good homogeneity; however, as β-stabilizing elements, both Cr and Nb favor β phase domains.

**Figure 10:**
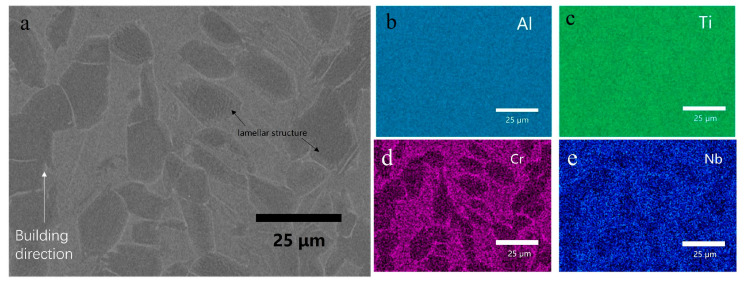
(**a**) BSE image of the HT sample at a higher magnification, EDS map of (**b**) Al, (**c**) Ti, (**d**) Cr, and (**e**) Nb.

Figure 10a shows a BSE image at a higher magnification. In the equiaxed α_2_ grains, some lamellar structures can be observed. In Figure 10d, the β phase region has a higher Cr content, and the α_2_ region contains limited Cr. In Figure 10e, Nb also shows a similar trend; however, the segregation is not as obvious as Cr, indicating that Nb exists not only in the β phase but also in the α_2_ phase with a small amount.

**Figure 11:**
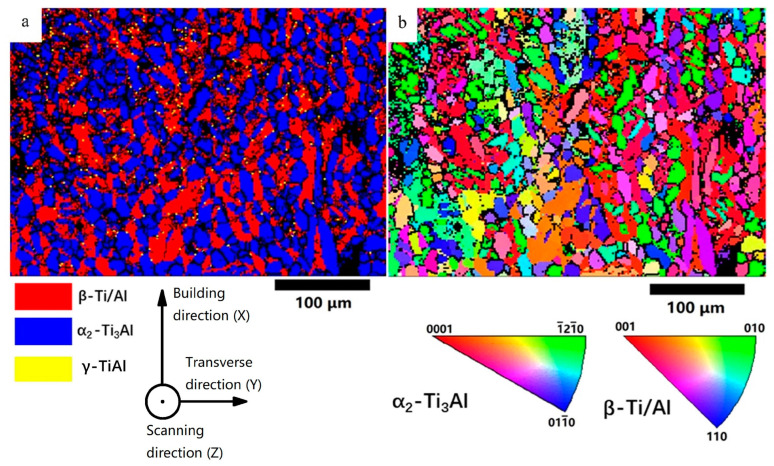
(**a**) EBSD phase distribution image of the HT sample, (**b**) inverse pole figure of the HT sample.

To understand the recrystallization behavior of the α_2_ grains, EBSD was carried out, as shown in Figure 11. Figure 11a shows the phase map, confirming the equiaxed fine grains consist of α_2_ phase and the matrix is β phase. The average grain size of the α_2_ phase was measured to be 15 ± 4.7 μm, and the average size of β grains was 19.4 ± 9.5 μm. α_2_ phase contained a 60.3% area fraction of the indexed region, and the β phase contained 39.7%. 

**Figure 12:**
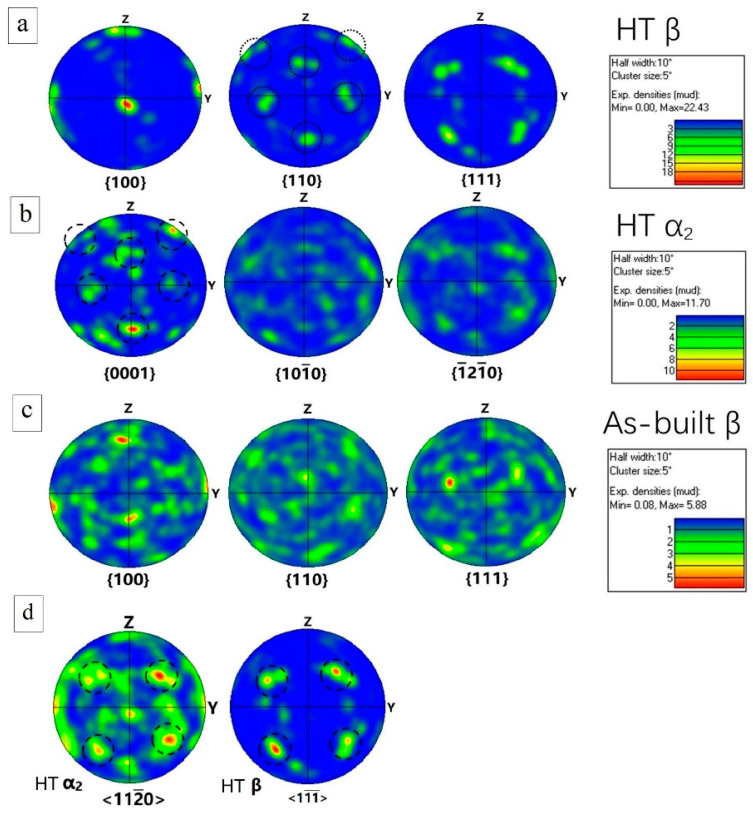
The pole figure of (**a**) HT β phase sample, (**b**) HT α_2_, (**c**) as-built β, (**d**) HT α_2_<11$\overset{-}{2}$0> and HT β<1$\overset{-}{11}$ >, showing the texture evolution after the heat treatment.

Figure 12a,b show the pole figures of the β and α_2_ phases in the HT Ti_3_Al sample, respectively. In contrast, Figure 12c illustrated the pole figure of the β phase in the as-built Ti3Al sample. In the as-built sample, the β phase does not show a strong texture (MUD = 5.88), however after the HT, a strong <100> fiber texture (MUD = 22.43) with a small mismatch angle of ~10 ° from the building direction was found. This crystallographic texture is widely reported in the thin structure LPBF of the materials with a cubic crystal structure [46,47]. The fact that the fiber texture was not strong in the as-built sample can be partially attributed to the presence of the partially-melted particles, which hinders the epitaxial growth of the β grains. During the HT, the partially-melt particles dissolve in the matrix at the temperature above the β-α transit (~1170 °C, according to the equilibrium phase diagram [48]). Comparing the pole figure of HT α_2_{0001} with HT β {110}, some crystallographic orientations align (labeled with dashed circles), which follows the Burgers orientation relationship (BOR), namely (0001)_α_ // (110)_β_, <11$\overset{-}{2}$0>_α_ // <1$\overset{-}{1}\overset{-}{1}$>_β_ [49]. The pole figures of HT α_2_ <11$\overset{-}{2}$0> and HT β <1$\overset{-}{1}\overset{-}{1}$> in Figure 12d also revealed this relationship. As far as the authors’ knowledge, this is the first time that BOR was observed between α_2_ and β phase in a Ti_3_Al alloy. α_2_ phase nucleates at the β grain boundaries, obeying BOR, and precipitates preferentially along with its {1000} pole, resulting in this observation by the pole figures. 

**Figure 13:**
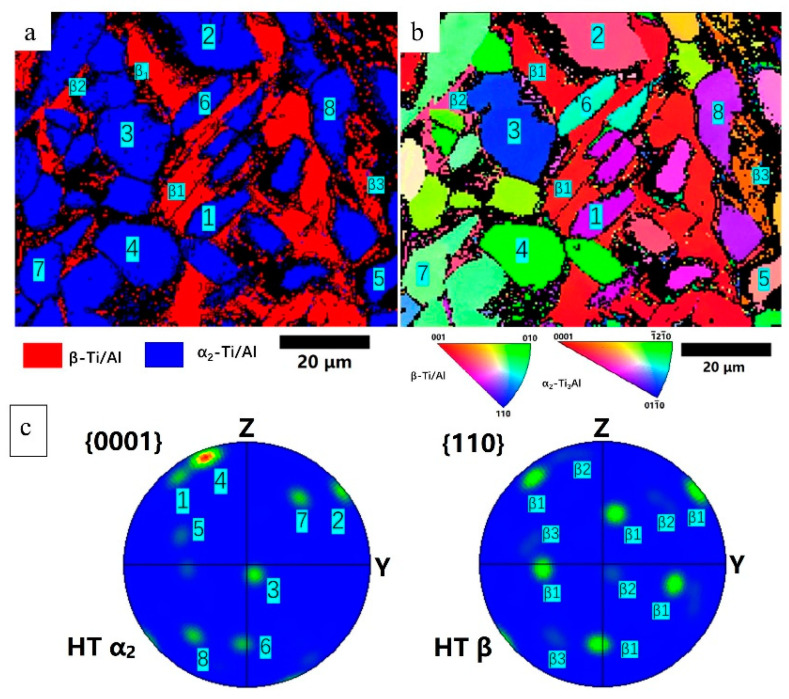
The orientation relationship in the HT sample: (**a**) phase distribution, (**b**) IPF, (**c**) pole figure of HT α_2_ {0001} and HT β {110}.

Figure 13a,b show the phase map and IPF of a region in the HT sample with a higher magnification, and Figure 13c shows the pole figures of HT α_2_ {0001} and HT β {110}. It can be observed that the {0001} planes of α_2_1, α_2_2, and α_2_6 are parallel to the {110} planes of β1, {0001} planes of α_2_3, α_2_4, and α_2_7 are parallel to the {110} planes of β2, and the {0001} planes of α_2_5 and α_2_8 are parallel to the {110} planes of β3. Respecting the Burgers orientation relationship, 12 hexagonal variants can be generated based on the parent β phase. Each α_2_ precipitation selected a preferential orientation on the grain boundaries of β1, β2, and β3 following BOR, and maintain the common {0001} pole in each α_2_ grain. This transformation mode has been repeatedly reported in the near-α titanium alloys [50,51], as a valuable understanding in controlling the crystallographic orientation and microstructure via HT [52,53]. Another preferential orientation selection of the α phase in near-α titanium alloys has also been reported, which is related to the pre-existed α [50]. Due to the presence of partially-melted CP-Ti particles (consisting of α phase) in the as-built sample, this mode should also be considered possible; however, this mode is hardly proved in the present study, which requires more detailed investigation in the future. 

### 3.4. Microhardness

**Figure 14:**
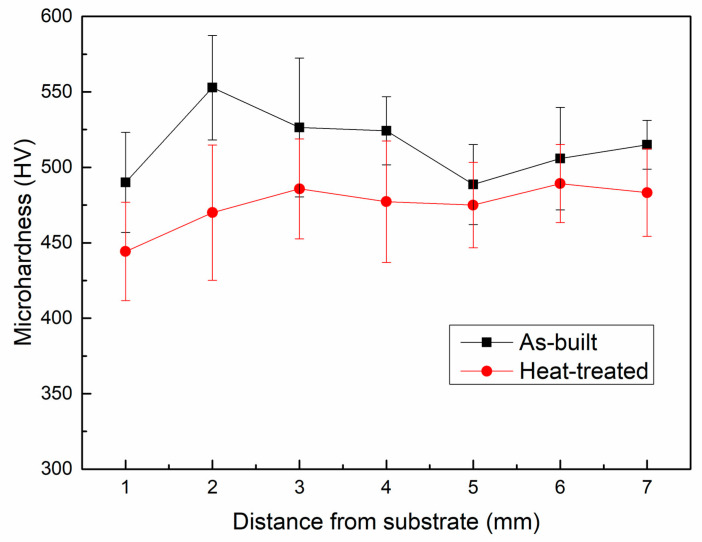
Microhardness of the as-built sample and heat-treated sample.

Microhardness was tested for the as-built and HT samples to exhibit the mechanical property, as illustrated in Figure 14. The microhardness values of different heights in the as-built sample are relatively similar, and the highest values appeared at 2 mm from the substrate. After the HT, the microhardness values of different heights all decreased. The as-built sample and HT sample showed the average microhardness of 515 ± 38 HV and 475 ± 37 HV, respectively, as presented in Table 2. This decrease in the HT sample can be contributed to both the high dislocation density in the as-built sample and the high heat treatment temperature used in this study. Typically, the LPBF process generates a huge number of dislocations in the as-built sample, which could lead to very high strength and hardness, along with low ductility [54,55]. After the heat treatment, the dislocation density decreases, and thus the ability to accommodate the deformation increases. As a result, the ductility increases, with a reduction in strength and hardness [55]. On the other hand, the HT in this study mainly aimed to dissolve the partially-melt particles in the as-built sample. Therefore, the HT temperature was relatively high, which is above the β transus. Typically, this HT condition leads to an increase in ductility but a decrease in strength and hardness [56]. Microhardness results from the previous studies regarding titanium aluminide intermetallics, also shown in Table 2, which showed inferior microhardness values compared with those of the as-built and HT samples in the present research. The values vary from 332 ± 18 HV to 510 HV, with different processing conditions, where the composition, processing condition, corresponding phase constituent, and microstructure affect the values. This comparison shows a promising research value of the method in this study in terms of microhardness. 

## 4. Conclusions

In this study, a crack-free fabrication of Ti_3_Al-based alloy was performed by LPBF with a powder blend, providing fundamental research on the Ti_3_Al lattice structure by LPBF. With the starting feedstock of CP-Ti and TiAl-4822 powder blends, the as-built strut sample showed good chemical homogeneity in the central region with some partially-melted particles in the peripherical regions. With the aid of the high cooling rate, a high volume fraction of the β phase was achieved to lower the cracking susceptibility. After the heat treatment of 1200 °C for 4 h, the partially-melted Ti particles were fully dissolved, and an α_2_/β dual-phase structure was achieved. The equiaxed α_2_ grains contained a volume fraction of 60.3%, with a grain size of 15 ± 4.7 μm. The precipitation of α_2_ grains from β grains followed the Burgers orientation relationship. The microhardness was measured to be 515 ± 38 HV in the as-built sample. After the heat treatment of 1200 °C for 4 h, the microhardness changed to 475 ± 37 HV. This study demonstrated a method to fabricate crack-free intermetallic components, which was considered inapplicable to LPBF, using a powder blend. Future studies in this field can be the lattice structure components fabrication, the scaling up of the thin strut, and the investigation of the effect of heat treatment, etc. 

## Figures and Tables

**Table 1:** D_10_, D_50_, and D_90_ of TiAl-4822, CP-Ti, and the powder feedstock mixture.

	D_10_ (µm)	D_50_ (µm)	D_90_ (µm)
TiAl-4822	25.36	33.9	45.27
CP-Ti	27.44	40.06	56.40
Mixture	25.36	36.05	53.47

**Table 2:** Microhardness of some Ti_3_Al alloys.

Composition	Method	Phase	Hardness (HV)	Reference
Ti-23Al-17Nb (at.%)	Linear friction welding	β, α_2_, O ^1^	448	Li et al. [57]
Ti-24Al-11Nb (at.%)	Hot roll + quenching	β/B2, α_2_	443	Suwas et al. [30]
Ti-22Al-12Nb (at.%)	Arc melting + hot vacuum pressing	B2, α_2_	510 (Arc melting) 392 (hot pressing)	Kumar et al. [42]
Ti-22Al-25Nb (at.%)	EB ^2^ welding	α_2_, O, B2	335 (as-weld), 420 (HT)	Chen et al. [58]
Ti-10Al-27Nb (wt.%)	EB welding	B2	342	Feng et al. [20]
Ti-22Al-25Nb (at.%)	LPBF ^3^ + HIP ^4^	α_2_, O, B2	332 ± 18	Polozov et al. [19]
Ti-22Al-25Nb (at.%)	LPBF + HT	α_2_, O, B2	338.6 ± 7.4 (as-built) 358.1 ± 5.8 (HT)	Grigoriev et al. [18]
Ti-33Al-1.4Cr-1.4Nb (at.%)	LPBF	β (as-built), α_2_ + β (HT)	515 ± 38 (as-built), 475 ± 37 (HT)	This study

^1^ O represents the orthorhombic phase [1]; ^2^ EB represents electron beam; ^3^ LPBF represents laser powder bed fusion; ^4^ HIP represents hot isostatic pressuring.

## Data Availability

Data will be made available on reasonable request.
